# Factors That Impact the Adoption of Clinical Decision Support Systems (CDSS) for Antibiotic Management

**DOI:** 10.3390/ijerph18041901

**Published:** 2021-02-16

**Authors:** Mah Laka, Adriana Milazzo, Tracy Merlin

**Affiliations:** 1 School of Public Health, University of Adelaide, Adelaide 5005, Australia; mah.laka@adelaide.edu.au (M.L.); adriana.milazzo@adelaide.edu.au (A.M.); 2 Adelaide Health Technology Assessment (AHTA), School of Public Health, University of Adelaide, Adelaide 5005, Australia

**Keywords:** clinical decision support tools, barriers, facilitators, UTAUT

## Abstract

The study evaluated individual and setting-specific factors that moderate clinicians’ perception regarding use of clinical decision support systems (CDSS) for antibiotic management. A cross-sectional online survey examined clinicians’ perceptions about CDSS implementation for antibiotic management in Australia. Multivariable logistic regression determined the association between drivers of CDSS adoption and different moderators. Clinical experience, CDSS use and care setting were important predictors of clinicians’ perception concerning CDSS adoption. Compared to nonusers, CDSS users were less likely to lack confidence in CDSS (OR = 0.63, 95%, CI = 0.32, 0.94) and consider it a threat to professional autonomy (OR = 0.47, 95%, CI = 0.08, 0.83). Conversely, there was higher likelihood in experienced clinicians (>20 years) to distrust CDSS (OR = 1.58, 95%, CI = 1.08, 2.23) due to fear of comprising their clinical judgement (OR = 1.68, 95%, CI = 1.27, 2.85). In primary care, clinicians were more likely to perceive time constraints (OR = 1.96, 95%, CI = 1.04, 3.70) and patient preference (OR = 1.84, 95%, CI = 1.19, 2.78) as barriers to CDSS adoption for antibiotic prescribing. Our findings provide differentiated understanding of the CDSS implementation landscape by identifying different individual, organisational and system-level factors that influence system adoption. The individual and setting characteristics can help understand the variability in CDSS adoption for antibiotic management in different clinicians.

## 1. Introduction

Antibiotic resistance has been recognised as a top-five major global health threat, and, by 2050, drug-resistant infections could lead to 10 million deaths worldwide [1]. Standardising clinical practice, improving the quality and safety of care and reducing inappropriate prescribing have become priorities for antimicrobial stewardship [2,3].

Clinical decision support systems (CDSS) are a digital health technology that provide clinicians with information at the point-of-care. By connecting evidence-based information on appropriate antibiotic prescribing with patient information, these systems filter and present accurate, real-time information to assist clinical decision making [4,5]. Benefits of CDSS for antibiotic stewardship include optimising the prescribing process by auditing decisions and providing real-time feedback, as well as increasing compliance with antibiotic prescribing guidelines and reducing the risk of unnecessary and inappropriate prescribing of specific antibiotics [6,7]. There is varying evidence available on the efficacy of CDSS for antibiotic management, but some studies have suggested that there can be reductions in the duration of antibiotic therapy, length of hospital stays, cost of antibiotic therapy and in-hospital mortality after the implementation of CDSS [6,8,9,10].

Studies have also shown that the availability of CDSS does not guarantee optimal adoption of the system by end-users. As a consequence, despite increasing evidence regarding CDSS benefits, CDSS adoption by end-users remains limited [11]. In healthcare organisations with CDSS in place, adoption is less than anticipated with 96% of CDSS alerts or recommendations usually overridden or ignored [12,13,14] for reasons attributed to end-users’ negative attitudes, evasion or scepticism regarding the system, as well as the unanticipated consequences of CDSS on clinical workflows [15,16,17].

The healthcare environment is characterized by an array of interdependent factors including clinical culture, processes, workflows and professional norms which can impact the successful introduction of systems such as CDSS [18]. A study conducted by Yusof et al. established that implementation of CDSS can be challenging due to the complex interaction of system, organisational and human factors [19]. Due to this complexity, it is difficult to ensure that improvement in one particular domain does not result in unanticipated consequences in another aspect of the care process.

CDSS implementation is also complicated because its scope extends far beyond a traditional information technology tool and integrates an evidence-based paradigm into every day clinical practice [20]. Liberati et al. report that scientific evidence provided by the system can sometimes challenge deep-rooted beliefs concerning professional autonomy and hierarchies of authority in the clinical setting, resulting in scepticism regarding the use of CDSS [13]. Many studies have focused on technical appropriateness and users’ experience to understand factors related to CDSS adoption [21,22,23], but there is limited information on how end-users’ individual characteristics influence perceptions about adopting CDSS for antibiotic management.

Our aim was to identify the different individual, organisational and system level factors that influence the adoption and use of CDSS for antibiotic management. This included identifying different individual- and setting-specific factors that moderate the perceptions of end-users. In doing so, we aim to establish a more differentiated understanding of the CDSS implementation landscape for antibiotic management in different settings. This information will be key to understanding the dynamics of CDSS implementation and identify underlying reasons for variation in CDSS adoption by clinicians.

## 2. Materials and Methods

### 2.1. Theoretical Framework

We used the unified theory of acceptance and use of technology (UTAUT) model to understand the interplay between different organisational, individual and technical factors influencing adoption and use of CDSS for antibiotic management [24]. Denktash et al. identified that the majority of information technology adoption models offer similar constructs to explain technology acceptance behaviour. Researchers tended to choose feasible elements from these models thereby reducing the overall breadth and depth of the favoured framework. To overcome this, eight of the most commonly used models were integrated into the UTAUT to provide a comprehensive framework for the behavioural intent to adopt and use technology [25]. The UTAUT model comprises of four main constructs that impact technology adoption: effort expectancy, performance expectancy, social influence, and facilitating environment. Effort and performance expectancy are related to the quality of system design in terms of ease of use, integration into a normal workflow and perceived benefits for improving the performance. Social influence reflects the effect of social networks in an organisation to shape users’ behaviour to adopt and use any technology. The last construct of this framework, the facilitating environment, captures the users’ belief that any setting or organisation has an appropriate structure in place to sustain use of the technology. The environment may not impact the users’ intentions, but it directly influences the actual technology adoption and use. One of the key aspects of UTAUT is integration of user-specific factors that moderate the impact of model constructs. These moderating variables, including age, gender, and experience, influence the direction and magnitude of the effect of model constructs on the behavioural intent and actual use of technology.

**Figure 1:**
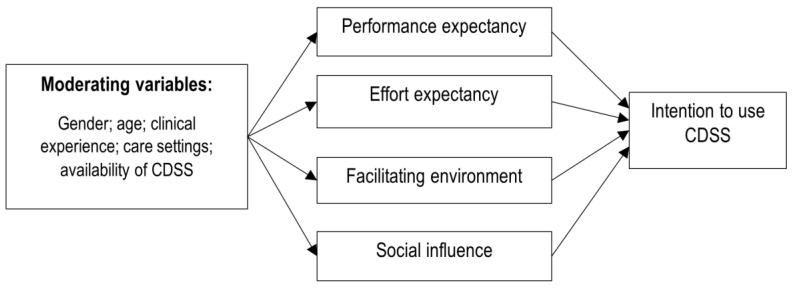
Theoretical framework of the study, the unified theory of acceptance and use of technology (UTAUT) model.

Figure 1 provides the study theoretical framework based on the UTAUT model.

### 2.2. Study Design

This study employed a cross-sectional descriptive design. An online survey of physicians, surgeons and general practitioners across Australia was administered through Survey Monkey ^TM^ (www.surveymonkey.com (accessed on 15 June–30 October 2019), San Mateo, CA, USA) from June–October 2019. Recruitment was assisted by the Royal Australasian Colleges of Physicians, General Practitioners and Surgeons and local health networks. The survey was promoted via their newsletters, websites, and social media accounts. We utilised the checklist by Kelley et al. [26] as a standard guide for the development, analysis and reporting of the survey.

### 2.3. Study Participants

Our survey population was hospital and primary care clinicians in Australia who are directly involved in antibiotic prescribing. The sampling framework used information from the National Health Workforce Data Set comprising medical practitioner data (2015–2018). With a 5% margin of error and a 95% confidence interval, we estimated we would need a sample size of 350 clinicians from primary care, and hospitals to generalise the results to all Australian clinicians. However, we also knew that this would be difficult to achieve, with low response rates common for clinician surveys [27,28,29].

### 2.4. Questionnaire Instrument

The survey questionnaire provided in the Supplementary S1 was designed following an extensive literature review of similar studies [9,15,22,23,30,31,32,33,34,35,36,37]. Supplementary S2 (supplementary table) outlines the studies from which the questionnaire constructs were derived. Questions regarding perceived benefit, barriers and facilitators of CDSS adoption could be answered using five-point Likert-type scales, where 1 represented “Strongly Disagree” and 5 = “Strongly Agree”. Most survey questions were closed, except for one open-ended question and comments section.

### 2.5. Piloting

The online survey was pilot tested with known clinical contacts (*n* = 10) to identify any potential problems in the survey questionnaire before it was widely distributed. After reviewing the results in the pilot phase, modifications were made to the survey’s text.

### 2.6. Measures

As it was not mandatory for participants to provide a response to all questions, the number of responses to each question was calculated separately.

Responses to the questions tended to cluster at the ends of each Likert five-point scale, so dichotomous dependent variables were generated by collapsing the responses of “Agree” and “Strongly Agree” into “Yes” and “Disagree” and “Strongly Disagree” into “No/Unsure”. The neutral response of “Neither Agree nor Disagree” was included in the “No/Unsure” category as the low responses meant it could not be included as a separate category. The rationale of combining neutral with negative responses was that the focus of the analysis was in identifying participants who positively or negatively responded to the survey questions.

### 2.7. Statistical Analysis

For analysis, the moderating factors (gender, age, clinical experience, care settings) and use of CDSS were considered as independent or predictor variables whereas perceived benefits, barriers and facilitators to use of CDSS were analysed as dependent variables. To evaluate the association between these dependent and predictor variables, we used multivariable logistic regression. The results were provided as an odds ratio (OR) with 95% confidence interval (CI). Statistical analysis was performed using Stata 15 (StataCorp LLC, College Station, TX, USA).

### 2.8. Qualitative Analysis

An open-ended question was used to obtain information on any specific concern(s) participants had about CDSS. The responses were categorised using a thematic analysis approach described by Braun and Clarke [38] using NVivo12 (QSR International Pty Ltd., Doncaster, Victoria, Australia). Preliminary codes were generated through open coding of the qualitative data [39]. Using recursive comparison, these codes were then refined and merged into conceptual themes.

### 2.9. Ethics Approval

Ethical approval was obtained from the University of Adelaide Human Research Ethics Committee (approval number: H-2019-094). Participation was voluntary and the data collected was nonidentifiable. To offset the expected low participation, the respondents were given the opportunity to participate in a draw either to win an iPad or equivalent donation made to the Hospital Research Foundation in recognition of their participation.

## 3. Results

### 3.1. Characteristics of Study Participants

A total of 180 clinicians participated in the survey with 74 from primary care and 106 from hospitals. Missing values for questions ranged from 5.1% to 13.3%. Participant demographic characteristics are described in Table 1.

### 3.2. Perceived Benefit of CDSS

Respondents had access to a variety of electronic systems/modules in their respective practices, with 52% having some form of CDSS available. Access to CDSS was higher in hospitals (58%) than in primary care (42%). Predefined order sets (57%) and alerts (49%) for antibiotic management were common features available to CDSS users. Conversely, CDSS did not provide specific functionality for antibiotic stewardship for 31% of respondents in our study.

**Figure 2:**
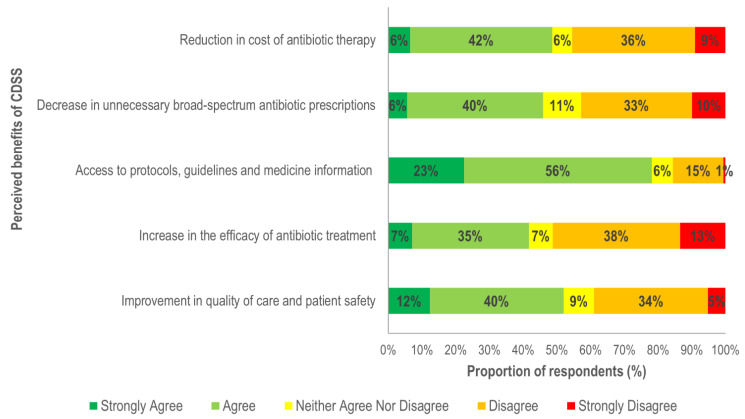
Overall perceived benefits of clinical decision support systems (CDSS).

**Figure 3:**
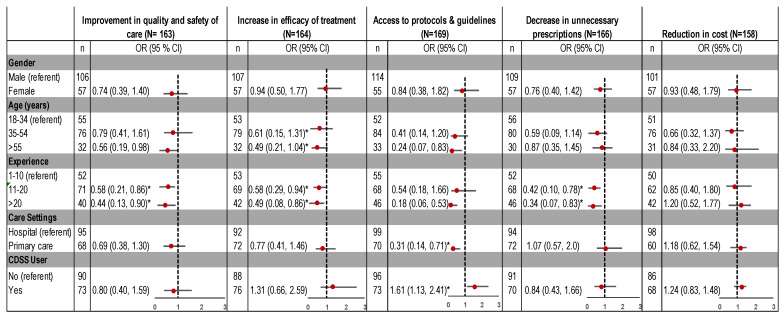
Association of perceived benefits to implementation of CDSS, by demographic characteristics. OR: odds ratio; CI: confidence interval. * Significant predictors, as confidence interval does not include 1.0.

In terms of perceived benefits, respondents (79%) agreed that CDSS implementation can increase accessibility to information for antibiotic management (Figure 2). CDSS users were 61% more likely than nonusers to believe that it can improve access to guidelines and care-related protocols (Figure 3). Clinicians in primary care were 69% less likely to recognise this benefit which may be related to the higher proportion of CDSS users in hospitals in our data.

Approximately half (52%) of the participants agreed that CDSS use is associated with improvements in the quality of care and would decrease unnecessary antibiotic prescriptions (46%), although this view was held mostly by clinicians with limited clinical experience. Respondents with 11–20, and >20 years of clinical experience were 42% and 56% less likely to believe that CDSS can positively impact the quality and safety of care. Experienced clinicians were also 58% (experience 11–20 years) and 66% (experience >20 years) less likely to believe that CDSS use is associated with a decrease in unnecessary antibiotic prescribing (Figure 3).

### 3.3. Perceived Barriers

**Figure 4:**
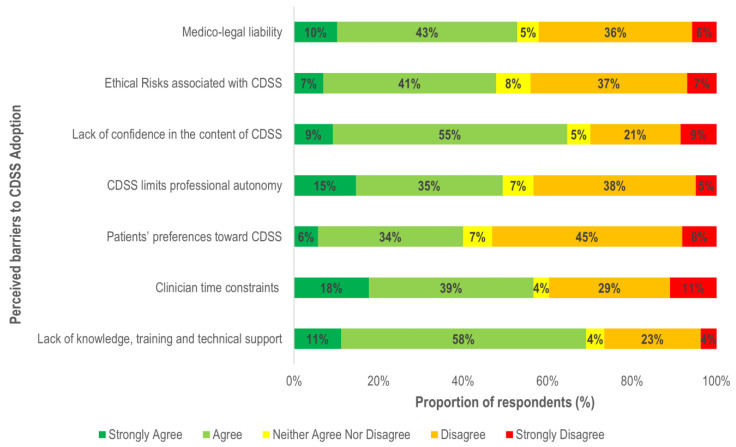
Reported barriers to CDSS adoption.

A lack of technical knowledge and training (69%) is an important barrier for CDSS adoption. Respondents (63%) also believed that end users’ lack of trust and confidence in the system’s content limits the usability (Figure 4).

**Figure 5:**
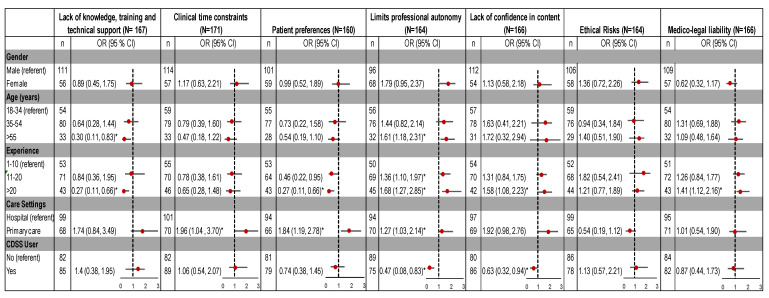
Association of perceived barriers to implementation of CDSS, by demographic characteristics. OR: odds ratio; CI: confidence interval. * Significant predictors as confidence interval does not include 1.0.

As shown in Figure 5, the type of healthcare setting was associated with clinician’s perceptions regarding barriers of time constraints, limits on professional autonomy, and patients’ expectations. Clinicians in primary care were more likely than those in hospitals to believe that factors such as time limitation (34%), threats to professional autonomy (27%) and patients’ preferences (84%) restrict the use of CDSS. Moreover, the likelihood of perceiving limited professional autonomy as a barrier was also found to increase with clinical experience (11–20 years: OR = 1.36, 95%, CI = 1.10, 1.97; >20 years: OR = 1.68, 95%, CI = 1.27, 2.85). Respondents in primary care (71%) were more likely to have >11 years clinical experience compared to those in hospitals (54%). Therefore, the association of settings with a threat to professional autonomy as a barrier may be related to a higher proportion of experienced respondents in the primary care group. Overall, clinicians with >20 years of clinical experience were more likely to believe that a lack of confidence in the CDSS content (58%) and risk of medico-legal liability (41%) would inhibit its use.

### 3.4. Perceived Facilitators

**Figure 6:**
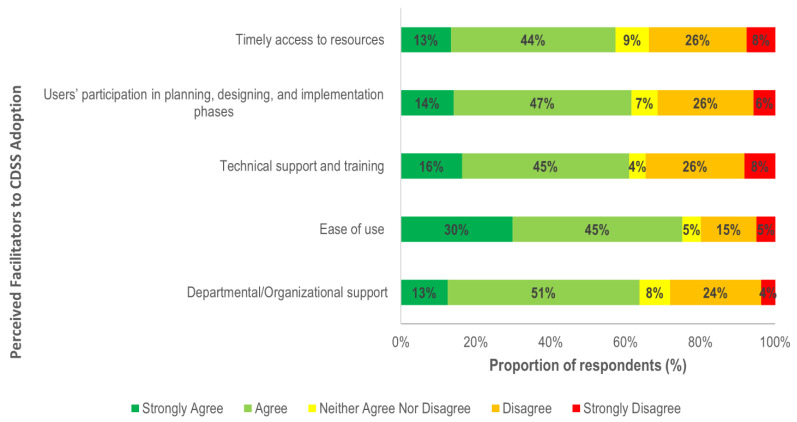
Facilitators to CDSS adoption.

Figure 6 highlights strong agreement (75%) of CDSS adoption if systems are easy to use, whereas 64% believed that organisational support is required for successful implementation. Along with organisational support, 61% also agreed that effective training and technical support ensures clinicians receive adequate support and skills to use it effectively.

**Figure 7:**
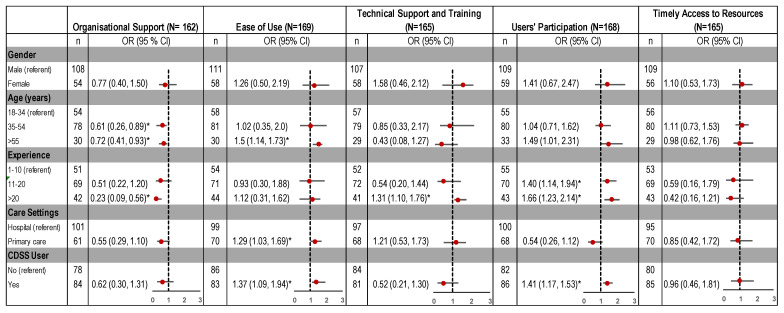
Association of perceived facilitators to CDSS implementation, by demographic characteristics. OR: odds ratio; CI: confidence interval. * Significant predictors as confidence interval does not include 1.0.

Healthcare setting, years of experience and CDSS use were associated with clinicians’ perception of different factors as enablers to CDSS adoption. Compared to hospitals, clinicians in primary care settings were 29% more likely to believe that ease of use will facilitate CDSS adoption (Figure 7).

Clinical experience (years) was also a significant predictor, with experienced clinicians more likely to believe that end-user consultation in the design and development of the system and the availability of technical support as important facilitators for use of CDSS. In comparison to non-users, there was higher likelihood in CDSS users to consider ease of use (OR = 1.37, 95%, CI = 1.09, 1.94) and users’ participation in the design and implementation phases (OR = 1.41, 95%, CI = 1.17, 1.53) as factors that enable adoption (Figure 7).

### 3.5. Qualitative Analysis

Analysis of free-text comments provided three major themes concerning factors that influence CDSS implementation:

i. Lack of Flexibility

Respondents expressed concerns regarding CDSS inflexibility to change as a barrier to adoption. System usefulness is significantly limited if it lacks the ability to reflect the complex clinical context by:

*“Systems I have experienced are comically bad in design mainly because they are inflexible in their ability to change.”*

(Hospital)

Clinicians require flexibility and adaptability in systems instead of “constant rule-making” to tailor recommendations to a specific context.

*“There is never a ‘one size fits all’. So there must always be room to make exceptions.”*

(Primary care)

ii. Information Overload

*“My major frustration with it [CDSS] in terms of antibiotic therapy is the presence of excessive alerts, which do nothing to protect patients and simply lead to alert fatigue.”*

(Hospital)

Information relevance and precision emerged as important factors influencing CDSS adoption. Excessive information with low specificity and relevancy leads to alert fatigue and the decision to override, thereby reducing the overall use of CDSS. Furthermore, it was highlighted that time and workload pressures make it difficult for clinicians to distinguish important information from irrelevant data.

iii. Information Accuracy

*“I, as a user, need to know on what basis any recommendation is provided, what is the source of this knowledge and how often it is updated.”*

(Primary care)

The accuracy of the content was also identified as an important theme for clinicians to trust the CDSS. Respondents expressed doubts concerning the currency and reliability of the content which then determines their overall trust in the system.

*“…[W]ithout knowing how often guidelines are updated in the system, we cannot rely on system alerts.”*

(Hospital)

The uncertainty felt by clinicians about the quality and accuracy of evidence negatively impacts their perception of CDSS.

## 4. Discussion

Our study contributes to the existing body of evidence by highlighting clinicians’ perceptions regarding CDSS implementation for antibiotic management. We focused on internal and external factors influencing users’ intent to adopt CDSS by incorporating the UTAUT framework. Internal related factors were specific to personal perceptions, whereas external factors represented organisational, technical or patient related factors. While previous studies have illustrated different factors determining users’ acceptance of CDSS related to antibiotic use, there is limited understanding of underlying factors that contribute to variations in acceptance of CDSS by different end-users. We addressed this gap in knowledge by evaluating the impact of age, gender, clinical experience, care setting and CDSS availability on users’ intention to adopt CDSS for antibiotic management.

### 4.1. Barriers and Facilitators

#### 4.1.1. External Factors

Lack of organisational capacity to provide appropriate technical support and training was a significant barrier and has been shown to limit users’ confidence in a system and the ability to resolve any technical issues that may arise, thus discouraging CDSS adoption [40,41]. Organisational theories also identify culture as an enabling factor for promoting the adoption of any new technology [42]. We found that young clinicians were more likely to require organisational support in order to adopt CDSS for antibiotic management, perhaps because clinical hierarchy and seniors’ preferences greatly influence the practices of young clinicians [43] and seniors’ in our study were less likely to adopt CDSS.

One of the basic system quality constructs of UTAUT is the ease of use. In our survey, ease of use was a key factor that facilitates the adoption and adherence of clinicians to CDSS use for antibiotic management. This is consistent with measures in the information system (IS) success model proposed by DeLone and Mclean (1992) that relates user satisfaction and adoption to ease of use [19,44]. We found that primary care clinicians and those with experience of using CDSS perceived ease of use to be one of the most important features for CDSS adoption. Limited consultation time, workload, and the potential for compromise of direct communication with patients due to the time required to navigate the system, make ease of use a highly relevant requirement for the successful implementation of CDSS in primary care [45,46,47].

System effort expectancy and perceived benefit is related to users’ trust that the system is a fit for their specific requirements [25]. Our results highlighted that clinicians with longer working experience tended to rate end-user consultation as an important facilitator for CDSS implementation. Similarly, our results also indicated that clinicians with longer clinical experience (>11 years) were more likely to see CDSS as a threat to their clinical autonomy. Therefore, inclusion of experienced clinicians in the CDSS development and implementation process would likely foster increased acceptance, trust and compliance with the system.

#### 4.1.2. Internal Factors

In our study, internal factors were frequently reported as barriers to CDSS adoption for antibiotic management, with lack of confidence in the content of the system most frequently reported. This was a common concern of CDSS nonusers in our study, which suggests that it could be a result of limited understanding of how the system sources information to guide recommendations, along with a lack of trust in personnel involved in system development, and lack of agreement with the content [48,49]. The apprehension that adoption of CDSS would compromise individual clinical judgements increases the reluctance of clinicians to engage with the technology [50,51]. Clinicians in our study with experience of using CDSS were less likely to believe that use of CDSS would compromise their professional autonomy, suggesting that end-user reluctance to adopt CDSS for these reasons might be the result of a perception about the system rather than actual experience with the system. Experienced clinicians were also less likely to use CDSS due to fear of compromising established work practices and reducing autonomous control over these processes and the content of clinical decisions. Studies indicate that younger clinicians tend to have better technological literacy and are more confident in using systems such as CDSS [11,52]. Our results are consistent with this literature, as a higher proportion of younger clinicians amongst our respondents were CDSS users as compared to senior clinicians. To overcome barriers to CDSS adoption, an effective clinical engagement process with experienced clinicians is required. The aim would be to empower early adopters amongst this cohort to drive the change process and advocate use of CDSS among their peers.

We found that clinicians’ time constraints and risk of workflow disruptions may also contribute to end-user resistance to CDSS adoption for antibiotic management. This is consistent with previous studies suggesting that failure to provide a fit between relevance, format and timeliness of recommendations negatively impact the uptake and utilisation of CDSS more generally [14,53]. Moreover, our findings also highlight that there is a greater likelihood in primary care of perceiving time and workflow constraints as barriers to CDSS adoption. Despite the fact that workflow disruptions and time sensitivities (high workloads) are relevant across all healthcare settings, the need to assess clinical data within a short consult might contribute to limited CDSS adoption in primary care [54].

Our findings highlight the impact of moderating factors, such as age, clinical experience and digital health literacy, shape clinicians’ behaviour in adopting digital health systems. These findings are consistent with the wider literature on the influence of these factors on the perceived usefulness and users’ intention to adopt other digital health systems [55,56,57]. For example, Jacob et al. recommended that understanding users’ inclination based on these moderating factors can help reduce the heterogeneity in system adoption and enable a cultural shift across all clinicians [58]. Future work should be directed toward establishing guidelines and a policy framework to address the barriers to CDSS adoption for antibiotic management. Our findings identified a range of individual and setting characteristics that influence the adoption and use of these types of CDSS. Further work in addressing organisational barriers and identifying optimal structures in terms of planning, management, leadership and communication to support CDSS implementation should be considered. Our study has several limitations. Because of low participation, the results may not be representative of the knowledge and perceptions of all Australian clinicians. Although the participation rate was not as high as planned, it is not dissimilar to other surveys conducted with clinicians [29,58,59]. Participants may have self-selected as a consequence of polarised views on the topic, introducing selection bias. Another limitation is the cross-sectional design of this survey limiting the ability to draw any causal inference. Also, respondents’ perceptions of factors related to CDSS for antibiotic management may not correlate with their actual practice. We allowed open responses to some questions which may have mitigated this to some extent.

## 5. Conclusions

This study advances the current knowledge of how different factors influence clinicians’ perceptions about CDSS adoption for antibiotic management. Comparisons between CDSS users and nonusers indicate that certain negative perceptions about CDSS for antibiotic management were related to a lack of clinical engagement and understanding of CDSS. Experienced clinicians were more likely to trust their own knowledge and approaches to prescribing antibiotics and were more sceptical of adopting CDSS. Similarly, time constraints and patient preferences in primary care were important factors in understanding clinicians’ reluctance to adopt CDSS for this purpose. Easy-to-use, flexible systems are more likely to be adopted, particularly if experienced clinicians are involved in their development, have confidence in the information and currency of the content in the CDSS and are trained in how to use the systems. These findings may help health service delivery organisations to successfully implement CDSS for antibiotic stewardship.

## Figures and Tables

**Table 1:** Characteristics of study participants.

Characteristics		*n* = 180 (%)
**Gender**		
Male		118 (66)
Female		62 (34)
**Age-Group**		
18–34 years		61 (34)
35–54 years		84 (47)
55 years and over		35 (19)
**Years of Experience**		
1–10 years		57 (32)
11–20 years		75 (42)
More than 20 years		48 (27)
Care setting and Type of Practice *		
Hospital(s)	Public	44 (24)
	Private	14 (8)
	Mixed	35 (19)
	Total	93 (51)
Primary care	Private	15 (8)
	Community clinic	11 (6)
	Hospital-based clinic	12 (7)
	Mixed	25 (14)
	Total	63 (35)
State and Territory, Australia (*n* = 139) *		
Eastern (ACT/NSW/QLD/TAS/VIC)		101 (73)
Central (SA/NT)		21 (15)
Western (WA)		17 (12)

* Non-mandatory question in the survey, thus, number not equal to total sample size (*n* = 180) due to missing value.

## Data Availability

The survey data is not publicly available due to restricted data consent provided by participants concerning sharing of information. A limited dataset is available from the corresponding author on request.

## Supplementary Materials

The following are available online at https://www.mdpi.com/1660-4601/18/4/1901/s1, Supplementary S1: Survey Questionnaire, Supplementary S2: Selection of questionnaire constructs.
