# Supplementary material for: Factors That Impact the Adoption of Clinical Decision Support Systems (CDSS) for Antibiotic Management
Source: Int J Environ Res Public Health. 2021 Feb 16;18(4):1901. doi: 10.3390/ijerph18041901 (PMC7920296; doi:10.3390/ijerph18041901)
Supplement: Supplementary file 1 [file ijerph-18-01901-s001.pdf]

Supplementary S1: Survey Questionnaire

Electronic Consent

Please note that completion of the survey indicates your consent to being involved in the study.

i. **What is your age, please select the appropriate age category**

☐ Below 18 ☐ 18-24 ☐ 25-34 ☐ 35-44 ☐ 45-54 ☐ 55-64
☐ Above 65

ii. **What is your gender?**

☐ Male ☐ Female ☐ Other

iii. **You have been practicing medicine for**

☐ 1-5 years ☐ 6-10 years ☐ 11-15 years ☐ 16-20 years ☐ > 20 years

iv. **Select the area of specialization**

☐ Medicine ☐ Surgery ☐ Paediatrics ☐ Gynaecology and Obstetrics ☐ Orthopaedics

☐ Urology ☐ Dermatology ☐ Ophthalmology ☐ Cardiology ☐ Others

v. **Select the type of your practice(s)**

☐ Public practice ☐ Private practice ☐ Mixed practice

vi. **Please enter the postcode of your workplace(s)**

____

vii. **Do you follow any specific antibiotic prescription guidelines in your practice?**

a. Yes
b. No
c. No information about antibiotic prescription guidelines

If yes, please specify whether these guidelines are:

a. Intranet/local guidelines
b. National guidelines (e.g. Therapeutic Guidelines: Antibiotics)

viii. **How often do you use the above specified guidelines for prescribing antibiotics?**

a. Multiple times in a day
b. Once a day
c. Once a week
d. Once a month
e. Never

ix. **In your opinion, which of the following factors can be perceived as barriers to appropriate antibiotic prescribing (please select all that apply):**

a. Delay in diagnostic tests/cultures
b. Lack of information (formulary restrictions, local resistance pattern etc.)
c. Absence of prescription guidelines
d. Pressure from patients
e. Knowledge and perceptions of healthcare providers
f. Others (please specify): ____________________________________

x. **Does your hospital have any electronic system/module for the following purposes?**

	No system	Basic system	Fully functional advanced system
a. Patient information management (i.e. electronic medical records)			
b. Medication management (i.e. electronic ordering)			
c. Decision support (i.e. alerts and reminders)			

xi. **If there is an clinical decision support system in your hospital(s), which of the following functionalities it provides specifically for antibiotic stewardship (please select all that apply):**

a. Optimal selection of treatment
b. Formulary information on antibiotic treatments
c. Protocols and antibiotic prescription guidelines
d. Knowledge-base (central repository of information)
e. Allergy/mismatch alerts

f. Prescription based-reminders
g. Dosing calculators
h. No functionality is specific to antibiotic stewardship
i. Any other: (please specify) ______________________________

xii. **In your opinion, what can be the likely impact of electronic decision support systems on antibiotic management in your hospital?**

	Strongly disagree	Disagree	Neutral	Agree	Strongly agree
a. Improvement in quality of care and patient safety					
b. Increase in efficacy of antibiotic treatment					
c. Access to protocols, guidelines and medicine information					
d. Decrease in unnecessary broad-spectrum antibiotic prescriptions					
e. Reduction in cost of antibiotic therapy					

xiii. **Do you think that the following factors can be considered as barriers for clinical decision support system?**

	Strongly disagree	Disagree	Neutral	Agree	Strongly agree
a. Lack of knowledge, training and technical support on					
b. Clinical time constraints					
c. Patients' preferences					
d. Limits professional autonomy					
e. Lack of confidence in the content of the system					
f. Ethical Risks associated with clinical decision support systems					

g.	Medico-legal liability

xiv. **Which of the following factors can facilitate the use of clinical decision support system in your daily practice?**

	Strongly disagree	Disagree	Neutral	Agree	Strongly agree
a. Departmental/Organizational support					
b. Ease of use					
c. Technical support and training					
d. Users' participation in planning, designing and implementation phases					
e. Timely access to resources					

xv. **Do you want to share any specific recommendation(s)/concern(s) for implementation of clinical decision support systems for antibiotic management?**

Supplementary S2 Selection of questionnaire constructs from the literature

	Factors	Sources
**Perceived Benefits**	Improvement in quality and safety of care	[1-3]
	Increase in efficacy of treatment	[2,4,5]
	Access to protocol & guidelines	[1,3]
	Decrease in unnecessary prescriptions	[2,5]
	Reduction in cost	[4]
**Barriers**	Lack of knowledge, training or support	[3,6-9]
	Clinical time constraint	[3,5,6,9]
	Limit professional autonomy	[4-7]
	Lack of confidence in content	[3,6,10]
	Ethical risks	[3,7]
	Medico-legal liability	[3,9]
**Facilitators**	Organisational support	[3,6,10,11]
	Ease of use	[1,3,5-8,11]
	Technical support & training	[3,6,8,9]
	Users' participation	[5-7,10]
	Timely access to resources	[5,7,10,11]

References

1. Goh, W.P.; Tao, X.; Zhang, J.; Yong, J. Decision support systems for adoption in dental clinics: A survey. *Knowledge-Based Systems* **2016**, *104*, 195-206.
2. May, L.; Gudger, G.; Armstrong, P.; Brooks, G.; Hinds, P.; Bhat, R.; Moran, G.J.; Schwartz, L.; Cosgrove, S.E.; Klein, E.Y.*, et al.* Multisite exploration of clinical decision making for antibiotic use by emergency medicine providers using quantitative and qualitative methods. *Infection control and hospital epidemiology* **2014**, *35*, 1114-1125.
3. Catho, G.; Centemero, N.S.; Catho, H.; Ranzani, A.; Balmelli, C.; Landelle, C.; Zanichelli, V.; Huttner, B.D. Factors determining the adherence to antimicrobial guidelines and the adoption of computerised decision support systems by physicians: A qualitative study in three european hospitals. *Int J Med Inform* **2020**, *141*, 104233.
4. Broom, J.; Broom, A.; Plage, S.; Adams, K.; Post, J.J. Barriers to uptake of antimicrobial advice in a uk hospital: A qualitative study. *Journal of Hospital Infection* **2016**, *93*, 418-422.
5. Chung, P.; Scandlyn, J.; Dayan, P.S.; Mistry, R.D. Working at the intersection of context, culture, and technology: Provider perspectives on antimicrobial stewardship in the emergency department using electronic health record clinical decision support. *American Journal of Infection Control* **2017**, *45*, 1198-1202.
6. Devaraj, S.; Sharma, S.K.; Fausto, D.J.; Viernes, S.; Kharrazi, H. Barriers and facilitators to clinical decision support systems adoption: A systematic review. *Journal of Business Administration Research* **2014**, *3*, p36.
7. Esmaeilzadeh, P.; Sambasivan, M.; Kumar, N.; Nezakati, H. Adoption of clinical decision support systems in a developing country: Antecedents and outcomes of physician's threat to perceived professional autonomy. *International Journal of Medical Informatics* **2015**, *84*, 548-560.
8. Rigopoulos, G.; Psarras, J.; Askounis, D. A tam model to evaluate user's attitude towards adoption of decision support systems. *Journal of Applied Sciences* **2008**, *8*, 899-902.
9. Short, D.; Frischer, M.; Bashford, J. Barriers to the adoption of computerised decision support systems in general practice consultations: A qualitative study of gps' perspectives. *International Journal of Medical Informatics* **2004**, *73*, 357-362.
10. Zheng, K.; Padman, R.; Johnson, M.P.; Diamond, H.S. Understanding technology adoption in clinical care: Clinician adoption behavior of a point-of-care reminder system. *International Journal of Medical Informatics* **2005**, *74*, 535-543.
11. Jung, S.Y.; Hwang, H.; Lee, K.; Lee, H.Y.; Kim, E.; Kim, M.; Cho, I.Y. Barriers and facilitators to implementation of medication decision support systems in electronic medical records: Mixed methods approach based on structural equation modeling and qualitative analysis. *JMIR Med Inform* **2020**, *8*, e18758.
